# Development and Validation of a Novel Five-Dye Short Tandem Repeat Panel for Forensic Identification of 11 Species

**DOI:** 10.3389/fgene.2020.01005

**Published:** 2020-09-24

**Authors:** Wei Cui, Xiaoye Jin, Yuxin Guo, Chong Chen, Wenqing Zhang, Yijie Wang, Jiangwei Lan, Bofeng Zhu

**Affiliations:** ^1^Key Laboratory of Shaanxi Province for Craniofacial Precision Medicine Research, College of Stomatology, Xi’an Jiaotong University, Xi’an, China; ^2^Clinical Research Center of Shaanxi Province for Dental and Maxillofacial Diseases, College of Stomatology, Xi’an Jiaotong University, Xi’an, China; ^3^College of Medicine and Forensics, Xi’an Jiaotong University Health Science Center, Xi’an, China; ^4^Multi-Omics Innovative Research Center of Forensic Identification, Department of Forensic Genetics, School of Forensic Medicine, Southern Medical University, Guangzhou, China

**Keywords:** species identification, meat fraud, developmental validation, forensic science, short tandem repeat

## Abstract

Species identification of unknown biological samples is of fundamental importance for forensic applications, especially in crime detection, poaching, and illegal trade of endangered animals as well as meat fraud. In this study, a novel panel was developed to simultaneously identify 10 different animal species (*Gallus domesticus*, *Anas platyrhynchos domesticus*, *Ovis aries*, *Sus scrofa domesticus*, *Bos taurus*, *Equus caballus*, *Columba livia domestica*, *Rattus norvegicus*, *Mus musculus*, and *Canis lupus familiaris*) and human beings by amplifying 22 short tandem repeat (STR) loci in a multiplex PCR using a set of five fluorescently labeled dyes. This novel 22-STR panel was validated by optimization of PCR conditions as well as species specificity, sensitivity, reproducibility, precision, DNA mixture, and tissue/organ consistency. The results of developmental validation showed that the 22-STR loci achieved high species specificity among 10 animal species and human beings, and the sensitivity of this panel was 0.09 ng. This 22-STR panel identified different meats in mixed samples, and the minimum detected mixture ratio in the current test was 10% (0.1 ng/1 ng). This sensitive, accurate, and specific 22-STR panel can be used for forensic species identification and the detection of meat fraud and adulteration.

## Introduction

Biological samples left behind at crime scenes always contain a great deal of valuable information that can provide helpful clues for the criminal investigations. In addition to human biological specimens, non-human biological samples acquired from a crime scene can also suggest certain directions for tracing the suspects. With the continuous progress of biotechnological achievements made in the field of forensic genetics, there has been much interest in the forensic identification of non-human species. For example, domestic pet hairs left at a crime scene could be evidence indicating that the suspect (pet owner) might have been present at the scene of the crime (Budowle et al., 2005).

Rhinoceros horn and tiger bone were two substances that were formerly used in traditional Chinese medicine. Although the production and import of these protective animal-derived traditional Chinese medicine are strictly prohibited in China, occasional illegal trade occurs. Species identification is then of great importance in the criminal investigation of poaching and illegal trade of endangered animals (Staats et al., 2016). Moreover, species identification of various animal utilizing genetic markers can be applied in the detection of species mislabeling, and the process also contributes to food safety by its utilization in meat adulteration cases (Iyengar, 2014).

With the growing size of the human population and increasing of social affluence in recent years, meat consumption has been increasing annually (Godfray et al., 2018). Along with the increase in meat consumption of different animals, meat fraud and adulteration events occasionally occur around the world. An example of this was the spread of a horsemeat scandal across Europe in 2013 (O’Mahony, 2013), which not only seriously undermined the market order but also increased the risk of religious and ethnic conflicts. Because there are now widespread meat fraud and adulteration, some effective measures should be taken to ensure the authenticity of meat products. Developing accurate meat identification techniques will play an important role in solving these problems mentioned above.

Based on the morphological and structural differences of cells and tissues in different species, the morphological observation was one of the most traditional techniques used for species identification a few decades ago (Lou et al., 2016). The serologic-based technique has been used for species identification since the 1980s. With relatively high accuracy and sensitivity compared with traditional morphology, serological methods, such as the colloidal gold test strip, have been used in species confirmatory tests (Matsuzawa et al., 1993). However, serology analysis is vulnerable to low antibody specificity or trace sample, as well as the ability to only distinguish between human and non-human specimens.

To date, the DNA-based species identification technique has been widely adopted as an effective molecular detection tool for the identification of non-human species due to the rapid development of polymerase chain reaction (PCR) and the high level of sensitivity achieved in recent years (Skouridou et al., 2019). DNA barcodes refer to a short DNA sequence from a standard locus which not only encompasses sufficient phylogenetic information to identify different species but is also easy to amplify and analyze (Nicolas et al., 2012). Currently, the most popular DNA barcodes are cytochrome *b* and cytochrome oxidase subunit 1, which are located on mitochondrial genome. Although there have been more than five million DNA barcodes published in the Barcode of Life Data System (BOLD)^1^, scientists found a severe lack of adequate taxonomic coverage of some animal species within BOLD, which might be the results of anomalous or invalid identification (Wilson-Wilde et al., 2010; Iyengar, 2014). However, an underestimation or overestimation of species DNA content might be a concluded when mixed samples were analyzed using mitochondrial markers based on real-time PCR (RT-PCR) or digital PCR (dPCR) due to the mitochondrial heterogeneity in different tissue or organs (Floren et al., 2015). Hence, DNA genetic markers located in the nuclear genome have increasingly become the promising molecular markers for animal species identification.

Appearing as a repeat unit of a 2–6 bp core sequence, the short tandem repeat (STR) loci, distributing widely in the human genome with high polymorphisms, have been widely used in the field of forensic genetics (Fordyce et al., 2015; Fang et al., 2018; Wang et al., 2018). In recent decades, the PCR-STR capillary electrophoresis (CE)-based technique has already matured and has been used as the gold standard method for individual identifications and kinship tests (Zhang et al., 2018).

In this study, we selected 22-STR loci of 10 different animals as well as human beings, with two STR loci for each species, which enabled high species specificity among pig, cattle, goat, chicken, duck, rat, mouse, horse, pigeon, canine, and human samples. And then, we constructed a novel five-dye typing panel based on the CE platform. To evaluate the forensic efficiency of this 22-STR panel, we conducted a series of validation tests such as sensitivity, species specificity, DNA mixture, reproducibility studies and so on.

## Materials and Methods

### Sample Collections and DNA Extraction

Samples of chicken (*Gallus domesticus*), duck (*Anas platyrhynchos domesticus*), sheep (*Ovis aries*), pig (*Sus scrofa domesticus*), cattle (*Bos taurus*), horse (*Equus caballus*), and pigeon (*Columba livia domestica*) were purchased in the local market. Samples of Sprague-Dawley (SD) rat (*Rattus norvegicus*), Kunming mouse (*Mus musculus*), and dog (*Canis lupus familiaris*) were acquired from the Medical Experimental Animal Center of Xi’an Jiaotong University. The environment of this center meets the standard for feeding practices conducted for experimental animals (GB 14925-2010). Human (*Homo sapiens*) blood stains were previously collected by our laboratory. SD rats and Kunming mice were anesthetized and euthanized by cervical dislocation. Euthanasia of the experimental animals, sample collections, and the following experimental processes were approved by the ethics committee of Xi’an Jiaotong University, Health Science Center.

DNA was extracted using a TIANamp Genomic DNA Kit (TIANGEN Biotech, Beijing, China). DNA was quantified with a NanoDrop 2000c spectrophotometer (Thermo Fisher Scientific, South San Francisco, CA, United States). If the concentration of extracted DNA was not greater than 1 ng/μl, the DNA was re-extracted.

### Selection of Species-Specific STR Loci and Primer Design

Twenty-two STR loci showing species specificity among 11 species were selected from published studies following the criteria: (1) primer sequences designed for each STR locus of one species did not share homologous sequences with other species; (2) priority was given to STR loci whose core sequences were tetranucleotide; and (3) priority was given to STR loci that had fewer alleles. Primer 5.0 software was used to design the STR primers. Oligo 7 software (Rychlik, 2007) was used to ensure that each primer was free from self-dimer and non-specific hybridization in other species genomic regions using Basic Local Alignment Search Tool (BLAST) provided by National Center for Biotechnology Information. Primers of 22 STR loci were commercially synthesized (Microread Genetics, Beijing, China). Four dyes were used to individually label these primers, and QD550 (orange, Microread Genetics, Beijing, China) was used to mark the internal size standard. Detailed information for each STR locus is shown in Table 1.

**TABLE 1:** Detail information of 22 STR loci in this novel panel for species identification.

Species	Locus	Chromosome	Accession	Repeat unit	Dye
Pig	EF046	13	NC010455.5	GT	HEX
	SW742	16	AF235351.1	GT	FAM
Cattle	BT165*	26	FJ232025	TATG	FAM
	BT150*	22	FJ232024	ATAC	TAMRA
Sheep	MAF33	OAR9/CHI14	M77200	CA	TAMRA
	MCM164	OAR2/CHI8	L39134	GT	HEX
Chicken	LEI0094	1	X83246.1	AC	HEX
	GCT025	2	AJ233970.1	(GAAA)_*m*_(GAAG)_*n*_(AAAG)_*o*_	FAM
Duck	APH14	Unknown	AJ272583.1	(CA)_*m*_A(CA)_*n*_	HEX
	CAUD056	Unknown	AY493301.1	TTTCCCTCTTTC	FAM
SD rat	D0UIA21	Unknown	AF053391	GATA	TAMRA
	D8UIA2	8	AF054019	GATA	TAMRA
Kunming mouse	NC000084	18	NC000084	TAGA	HEX
	NC000070	4	NC000070	GATA	HEX
Horse	HMS3	9	X74632.1	(TG)_2_(CA)_2_TC(CA)_*n*_ Or (TG)_2_(CA)_2_TC(CA)_*n*_GA(CA)_5_	TAMRA
	HMS6	4	X74635.1	GT	HEX
Pigeon	PG5	Unknown	Ref^#^	TTTG	FAM
	PG6	Unknown	Ref^#^	AAAC	FAM
Canine	FH2100	3	NC_006585.3	GAAT	ROX
	FH2361	29	FJ031001.1	GAAA	ROX
Human	D3S3045	3	NC_000003.12	GATA	TAMRA
	TPOX	2	M68651	AATG	ROX

*BT150 has changed to BT22, and BT165 has changed to BT26 in GenBank. ^#^These two STR loci were selected from Chun-Lee et al. (2007).

### Allelic Ladder Construction

For the 22-STR loci, 20 unrelated individuals of each species were collected to determine the variabilities of the alleles observed in each species. Moreover, variabilities of the alleles for some STR markers were screened from previously published studies. Allelic ladder was generated according to previous reports (Chen et al., 2019).

### Multiplex Amplification and Genotyping

Unless stated otherwise, standard PCR amplification and genotyping procedures were as follows. We used a 10-μl reaction volume containing 1 μl of DNA template (1 ng/μl), 2 μl of Primer set (Microread Genetics, Beijing, China), 4 μl of Master Mix I (Microread Genetics, Beijing, China), and 3 μl of deionized water. The PCR was conducted using a GeneAmp PCR System 9700 Thermal Cycler (Thermo Fisher Scientific, South San Francisco, CA, United States) under the following conditions: 5 min of initial denaturation at 95°C, followed by 29 cycles of 94°C for 30 s, 59°C for 60 s, and 72°C for 60 s, with the final elongation at 60°C for 60 min. Electrophoresis was performed by an ABI 3500xL Genetic Analyzer (Thermo Fisher Scientific, South San Francisco, CA) using 36-cm capillary arrays with POP-4^®^ Polymer (Thermo Fisher Scientific, South San Francisco, CA). Loading samples for CE contained 1 μl of PCR product, 0.3 μl of QD550 internal size standard, and 8.7 μl of Hi-Di Formamide. The alleles were genotyped using GeneMapper ID-X software v1.5 (Thermo Fisher Scientific, South San Francisco, CA, United States). Next, equal amounts of DNA from each species were mixed, and then the mixture was diluted to 1 ng/μl (DNA mix). The DNA mix was used as positive control DNA, and deionized water was used as the negative control.

### Construction of the Multiple Amplification STR Panel

#### Amplification of Each STR Locus

To evaluate the specificity and amplification efficiency of a pair of primers, we amplified each STR locus according to the standard PCR components and reaction conditions.

#### Optimization Study of PCR Conditions

PCR cycling parameter studies were conducted on the 10-μl volume system. Total cycle numbers of 27, 28, 29, 30, and 31 cycles, and annealing temperatures at 57, 58, 59, 60, and 61°C were separately tested in order to choose the most optimal PCR parameters.

#### Studies of the Total Volume of the PCR System and Uniformity of the PCR Amplification

Two different total reaction volumes, 10 and 25 μl, were adjusted to evaluate the performance of this 22-STR panel. The value of each reagent in the 25-μl PCR system was 2.5 times larger than that in the 10-μl system, and the PCR conditions were in accordance with those mentioned above. To evaluate the PCR performance in various PCR thermocyclers, we conducted the PCR in the Applied Biosystems Veriti Thermal Cycler, Applied Biosystems ProFlex Thermal Cycler, Applied Biosystems 9700 Thermal Cycler, and Applied Biosystems 2720 Thermal Cycler (Thermo Fisher Scientific, South San Francisco, CA). The DNA mix was regarded as the DNA template, and the reaction conditions used were as mentioned above.

### Developmental Validation Studies of This 22-STR Panel

#### Sensitivity, Reproducibility, and Precision Study

We performed a serial of concentrations of DNA mix to obtain 5 ng/μl, 2 ng/μl, 1 ng/μl, 0.5 ng/μl, 0.25 ng/μl, 125 pg/μl, and 62.5 pg/μl that were used to evaluate the sensitivity of this 22-STR panel. To evaluate the reproducibility of this panel, the DNA mix was genotyped three different times, comparing with the ladder profile. The mean sizes in base pairs and the standard deviations were calculated for each allele. We selected some samples of different species and sequenced the alleles of each STR locus using the Sanger sequencing method in order to verify the STR profile results of the CE platform. Sanger sequencing was conducted by Sangon Biotech^®^ Company (Sangon Biotech^®^ Co., Ltd, Shanghai, China). Genomic DNA was extracted from the liver, heart, spleen, lung, kidney, and muscle of the same SD rat and Kunming mouse, respectively, which was used to evaluate the concordance of genotyping results of this panel in detecting different organs or tissue of the same individual.

#### Specificity and Mixture Study

We genotyped DNA of each studied species based on this multiple STR panel to evaluate if the panel was capable of avoiding the genotype of other non-targeted species. Samples from practical cases were usually composed of more than one animal species, therefore, it was important to evaluate the reliability of this panel for the detection of mixture samples of different species. Mixture studies were also performed to evaluate the lowest detection limit (minimum amount of DNA/total amount of DNA) of this 22-STR panel. For this purpose, we set two types of DNA mixture patterns with different mix ratios, and these two mixture patterns are shown in Supplementary Table S1.

#### Casework Sample Verification

Human blood stains preserved on an FTA^TM^ card at room temperature for up to 7 years were used to represent common samples in the practical cases. We selected four unknown cooked meats to evaluate the efficiency of the detection of cooked meats. These four meats were named SE (braised), SY (roasted), SG (poached), and SN (stir-fried). The surface of all samples was rinsed with ultrapure water before DNA extraction to remove all inhibitory substances. All samples were amplified in a 10-μl volume, and the PCR conditions were the same as those mentioned above.

## Results

### Construction of This 22-STR Panel

In this research, 22-STR loci with high-species specificity among 10 animal species and human were selected: D8UIA2, D0UIA21; PG5, PG6; FH2361, FH2100; GCT025, LEI0049; HMS6, HMS3; BT165, BT150; D3S3045, TPOX; NC000070, NC000084; APH14, CAUD056; MAF33, MCM164; SW742 and EF046 loci.

Before the construction of this novel panel, we amplified each STR locus to evaluate the specificity and amplification efficiency of a pair of primers, and the results showed that specific peaks of each pair of primers were only detected at the corresponding locus for each species, and no peak was found in other species.

A series of temperatures at 57, 58, 59, 60, and 61°C were used to determine the optimal primer annealing temperature. All loci could be detected at these five different annealing temperatures, and the amplification efficiencies at 58, 59, and 60°C were higher than those at 57 and 61°C. The highest value of average peak height was observed when the annealing temperature was 58°C, but more optimal peak height balance was found at 59°C. We finally chose 59°C as the most optimal annealing temperature. Related genotype profiles are shown in Supplementary Figure S1.

**FIGURE 1:**
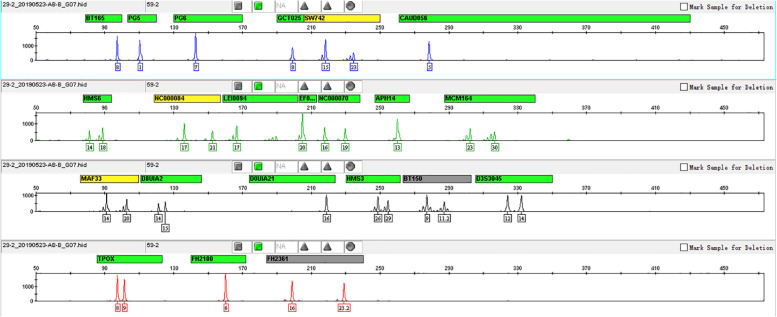
Genotyping profile of DNA mix amplified cycle numbers at 29 cycles. A more optimal peak height balance was observed with 29 cycles, and thus, we finally chose 29 as the optimal number of cycles.

This 22-STR panel was tested over a range of 27, 28, 29, 30, and 31 total amplification cycles. With the increase in the number of cycles, the peak height increased obviously. All alleles could be detected, and more optimal allelic peak height balance was obtained when the number of amplification cycles was 29. We finally chose 29 as the optimal number of amplification cycles. Related genotype profiles are shown in Figure 1.

We also evaluated the PCR efficiencies in the different reaction volume systems of 10 and 25 μl. As shown in Supplementary Figure S2, there was no allele drop in two systems, and higher peak heights were obtained in the 10-μl volume system as compared to the 25-μl volume system. We conducted the PCR using four different types of PCR machines, and the results showed that there was no obvious deviation of peak height balance among these four machines. The relative genotype profiles are shown in Supplementary Figure S3.

### Sensitivity, Reproducibility, Precision, and Concordance Study

**FIGURE 2:**
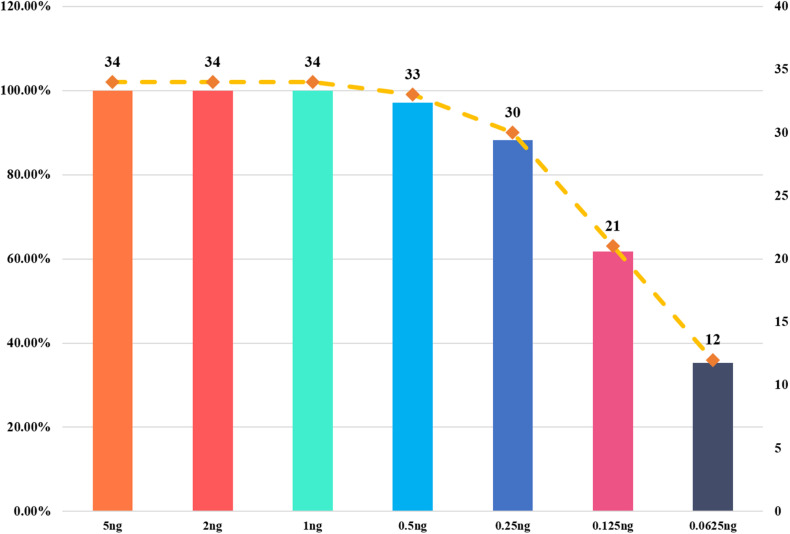
Sensitivity study of input DNA amounts ranging from 5 ng to 62.5 pg. Each bar represents the percentage of detected loci to total loci.

**FIGURE 3:**
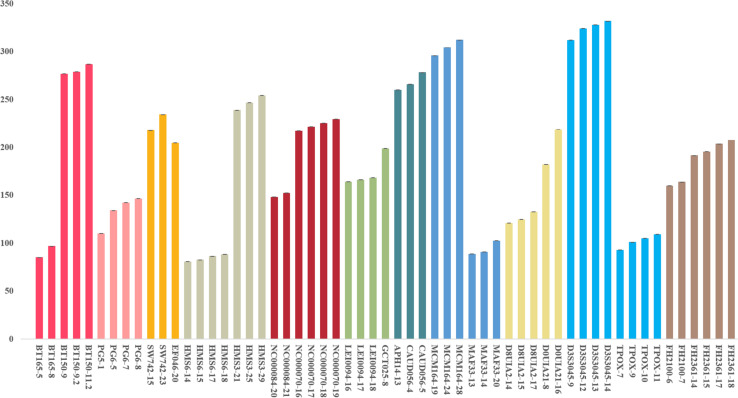
Allele size precision study of ladder. The bars represent the mean allele size of the ladder, and the error bars represent the plus and minus twice standard deviations in three experiments.

We used a serial of input DNA concentrations of 5 ng/μl, 2 ng/μl, 1 ng/μl, 0.5 ng/μl, 0.25 ng/μl, 125 pg/μl, and 62.5 pg/μl of DNA mix to evaluate the sensitivity of this 22-STR panel. As shown in Figure 2, small numbers of allele peaks dropped out when the template amount was 0.5 ng/μl, and many allele peaks dropped out when the template amounts were 125 or 62.5 pg/μl. Because the DNA mix contained equal amounts of DNA, the sensitivity of this panel was 0.09 ng (1 ng/11). The allelic ladder and DNA mix were genotyped three separate tests to evaluate the size precision. We measured the deviation of each allele size in these three experiments, and the results are shown in the bar chart in Figure 3. The bars in Figure 3 represent the mean allele size of the ladder, and the error bars represent the plus and minus twice standard deviations in three experiments. Figure 3 indicates that the standard deviation of each allele size is less than 0.1 bp. The results showed that in each sample, the allele sizes were consistent with their known amplicon sizes. We sequenced part of the alleles of each STR locus to evaluate the precision of the CE platform. The STR genotyping profiles acquired from the CE platform were consistent with the corresponding results of Sanger sequencing.

Genomic DNA extracted from the liver, heart, spleen, lung, kidney, and muscle of the same SD rat and Kunming mouse, respectively, were used to evaluate whether the STR genotyping profiles of different tissues or organs from the same individual showed exactly the same STR genotyping result. As shown in Supplementary Figure S4, allelic genotyping peaks could only be observed at the D8UIA2 (alleles: 14, 15) and D0UIA21 (alleles: 16, 16) loci when we co-amplified the genomic DNA extracted from different organs or tissue of a SD rat. For the Kunming mouse’s various organs or tissue (shown in Supplementary Figure S5), STR genotyping peaks could be detected at the NC000084 (allele: 17, 18) and NC000070 (allele: 17, 19) loci belonging to the Kunming mouse.

### Specificity, Mixture Study, and Casework Sample Verification

Genomic DNA templates of the studied species were amplified separately based on this multiplex STR panel so that we could evaluate the species specificity of this 22-STR panel, and the corresponding STR genotyping results are shown in Supplementary Figure S6. The profiles revealed that no peak was detected for the negative control. Specific allelic peaks were only detected at the corresponding loci for each species, and no allelic peak was found in other species-specific loci.

**FIGURE 4:**
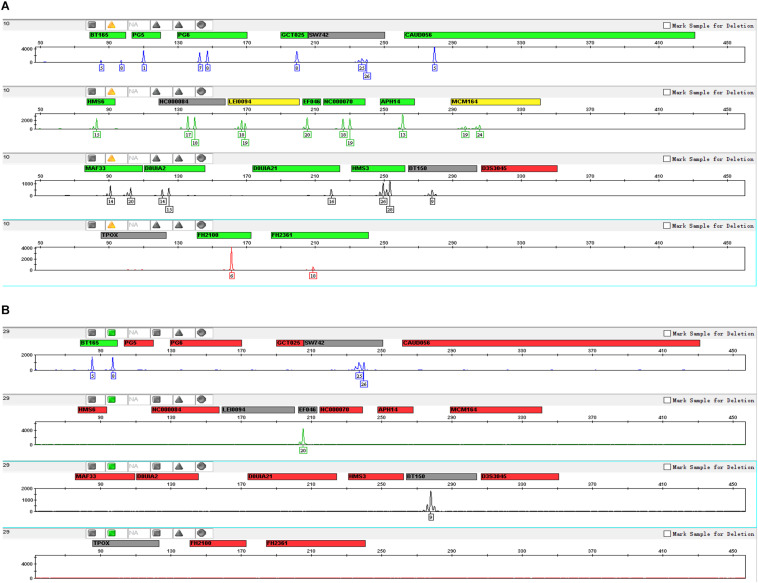
Mixture study of this 22-STR panel: **(A)** profile of DNA mixture of 10 species with the known ratio of 1:1:1:1:1:1:1:1:1:1 and a total of 1 ng of DNA; and **(B)** profile of a DNA mixture of pork and beef with the ratio of 3:1 and a total of 1 ng of DNA.

We made two types of DNA mix models with different mixture ratios to evaluate the performance of this panel in the detection of each DNA mixture. In Figure 4, we only displayed the genotyping profiles of DNA mixtures of 10 species (without human samples) with the known ratio of 1:1:1:1:1:1:1:1:1:1, and the DNA mixture of pork and beef with a ratio of 3:1. In these two types of mix models, all species were detected, and the detected ratio in the current test was 10% (0.1 ng/1 ng).

We genotyped human blood stains preserved for up to 7 years on FTA^TM^ cards at room temperature to evaluate the performance of this novel panel on aged samples. The results showed that all samples could be successfully genotyped at D3S3045 and TPOX loci.

We also genotyped four types of cooked meats of unknown species origin to evaluate the efficiency of the detection of cooked meats, and the profiles are shown in Supplementary Figure S7. Two pig-specific STR loci, SW742 (alleles: 15.1, 15.1) and EF046 (alleles: 20, 20), were observed in SG meat, indicating that SG was pork; two sheep-specific STR loci, MCM164 (alleles: 20, 28) and MAF33 (alleles: 13, 20), were observed in SY meat, indicating that it was mutton; two pig-specific STR loci, SW742 (alleles: 15.1, 24) and EF046 (alleles: 20, 20), were observed in SN meat, indicating that it was pork. LEI0094 was a chicken-specific STR locus that was observed in SE meat, but another locus, GCT025, was not detected, giving partial indication it was chicken.

## Discussion

With the increasing occurrence of illegal incidents such as meat fraud and adulteration or illegal trade of protected animals (Cawthorn et al., 2013; Tibola et al., 2018; LaFleur et al., 2019), it is fundamentally important in forensic genetics to be able to identify the animal species in an unknown sample. Compared to morphological observation and protein-based method, DNA-based method is regarded as one of the most suitable techniques for species identification due to their tolerance for heat or other environmental influences. To date, many panels for animal species identification have been developed on the basis of different DNA markers such as autosomal STRs (Dawnay et al., 2008; Liu et al., 2019), species-specific insertions-deletions (InDels) (Alves et al., 2017), and DNA barcodings (Arulandhu et al., 2017).

Over the past two decades, STR loci have been used extensively in population genetics, individual identification, and paternity tests for protected wild animals or domestic animals (Eiken et al., 2009; Gupta et al., 2011; Ogden et al., 2012; Wang et al., 2019). Despite their widespread use in the genetic research of non-human species, there were only a few STR-based panels used for the animal species identification.

Compared to human forensic genetics, research progress of non-human genetics has been more gradual, largely because no rich unified databases of wild animal or domestic animal were available. Besides, genetic markers used in the field of animal genetics have not been systematically validated by forensic medicine, which made them difficult to be used in the forensic genetics (Iyengar, 2014).

Most kits have been developed based on RT-PCR, dPCR, or liquid chromatography-tandem mass spectrometry (Floren et al., 2015; Kim et al., 2017; Xu et al., 2018). Although higher sensitivity and accuracy were acquired, the need for expensive instruments and their time-consuming operation made it difficult to apply these methods in the primary laboratories of China. At present, a PCR-STR-CE-based method is widely applied in most laboratories in China due to its relatively lower cost, higher efficiency, and mature technical system.

The purpose of this research is to develop a panel that could distinguish 10 animal species as well as human beings, which could then be used in the application of forensic species identification and the detection of meat fraud and adulteration. The choice of the studied species is fully considered based on actual adulteration cases. Pork, beef, mutton, and chicken are the most common meats found in China. Beef or mutton has been found to be adulterated with inexpensive meat such as duck, horse, and even mouse meat, and therefore, chicken, duck, sheep, pig, horse, cattle, rat, and mouse are selected for this study. Additionally, because canine and pigeon meat are also popular in some cities of China, these two animal species are chosen for this study.

We selected 22-STR loci with high species specificity among 11 species and then constructed a novel five-dye multiplex amplification panel that could be analyzed using the CE platform. Before the validations, we evaluated the performance of different thermal cycling parameters. As anticipated, an increasing cycle number led to an apparent increase in overall allelic peak height. All loci could be detected in reasonable ranges of thermal cycling parameters. At 29 cycles, we observed a more balanced peak height.

The annealing temperature affected the specificity of the PCR. In tests of different annealing temperatures, the amplification efficiencies at 58, 59, and 60°C were higher than those at 57 and 61°C. After we considered that low annealing temperature led to non-specific amplification (Rychlik et al., 1990), we finally chose 59°C as the optimal annealing temperature. After evaluating the PCR efficiencies in two reaction volumes, the results revealed that more optimal amplification occurred in the 10-μl volume (rather than 25 μl) containing 1 μl of DNA template, 2 μl of Primer set, 4 μl of Master Mix I, and 3 μl of deionized water.

It is essential to evaluate the efficiency of a novel panel before it is used for casework. Here, we performed a series of developmental validations studies including sensitivity, reproducibility, precision, specificity, mixture, and tissue/organ consistency and so on. In forensic practice, we could not always acquire sufficient DNA amounts, and therefore, any potential panel should be capable of genotyping trace amounts of DNA template. In the current study, we evaluated the sensitivity of this 22-STR panel with serial input DNA amounts. According to the results, one dropped peak (HMS3, allele 29) was observed when the input DNA was 0.5 ng, indicating that the minimum input amount of DNA template should be more than 0.5 ng. Reproducibility and precision studies were performed to validate the reliability and accuracy of this 22-STR panel. The results of reproducibility studies showed that the STR profiles of three trials were consistent, and allele calling was consistent with their known amplicon sizes, which demonstrated that this panel could ensure proper allele detection.

It was critical to ensure that this 22-STR panel exhibited no cross-reactivity between different species. The primer specificity of the STR loci was the key to the specificity of this panel. To ensure that no cross-reactivity occurred among the 11 species, we designed the primers according to the highly conserved region of each species’ genome and used BLAST to evaluate the specificity of each primer sequence. The present species specificity study showed that the specific peaks of the STRs were detected only at the corresponding loci for each species, and no allelic peaks were found in the STR loci of other species, indicating that all the primers in this panel exhibited no cross-reactivity between different species.

In the ongoing investigations of meat fraud and adulteration, it is usually found that various inexpensive meats such as chicken or duck are often added to beef or mutton, which not only decreases food safety, but also disrupts market order. Illegal addition of animal-derived ingredients in feedstuff might spread infectious diseases such as bovine spongiform encephalopathy or scrapie (Gao et al., 2017). Therefore, it is of fundamental importance to develop a panel with a high efficiency for the detection of the individual components in meat mixtures. According to the results of the mixture studies, all species in each mixture pattern could be detected, indicating that this panel would adequately function in the detection of mixed samples.

In addition to meat fraud and adulteration, processed meat or animal tissues are also commonly investigated in forensic casework. Poached and roasted lamb, beef, and pork are popular in the Chinese diet. High temperatures and various condiments used during cooking could damage DNA. Therefore, the efficiency of the detection of cooked meats or animal tissues is also essential. In this research, we used four different cooked meats to evaluate the detection efficiency of the mixture of cooked meat. Full profiles were acquired for SG, SY, and SN meats, and one locus was detected in SE meat. The results indicated that this 22-STR panel could be used for the detection of individual species in cooked meat.

## Conclusion

In this research, we developed a novel 5-dye panel that could simultaneously identify 10 animal species and human being, and co-amplify 22-STR loci using one PCR system. This panel was validated by a series of tests including optimization of PCR conditions, sensitivity, reproducibility, precision, species specificity, DNA mixture, and tissue/organ consistency. In present results, this 22-STR panel achieved high species specificity among 11 species and a detection capacity for a mixture of meat samples. The results of the developmental validations demonstrated that this panel can be used for forensic species identification and the detection of meat fraud and adulteration.

## Data Availability Statement

All datasets generated for this study are included in the article/Supplementary Material, further inquiries can be directed to the corresponding author.

## Ethics Statement

The studies involving human participants were reviewed and approved by the Ethics committee of the Xi’an Jiaotong University, Health Science Center. The patients/participants provided their written informed consent to participate in this study. The animal study was reviewed and approved by the Ethics committee of the Xi’an Jiaotong University, Health Science Center.

## Author Contributions

BZ designed and was responsible for this research. WC and XJ built up this 22-STR panel and prepared the preliminary data. WC, YG, and CC analyzed the data. WC wrote the draft manuscript. WZ, JL, YW, and BZ reviewed and revised the manuscript. All authors contributed to the article and approved the submitted version.

## Conflict of Interest

The authors declare that the research was conducted in the absence of any commercial or financial relationships that could be construed as a potential conflict of interest.

## Footnotes

1 http://www.barcodinglife.org/

## Supplementary Material

FIGURE S1 Genotyping profiles of annealing temperatures at 57, 58, 59, 60, and 61°C.

FIGURE S2 PCR efficiency studies of the two different reaction volume systems: 10 and 25 μl.

FIGURE S3 Genotyping profiles of PCR efficiency studies using four different types of PCR machines.

FIGURE S4 Genotyping profiles of DNA samples extracted from the liver, heart, spleen, lung, kidney, and muscle of the same SD rat.

FIGURE S5 Genotyping profiles of DNA samples extracted from the liver, heart, spleen, lung, kidney, and muscle of the same Kunming mouse.

FIGURE S6 Genotyping profiles of species specificity studies on this 22-STR panel.

FIGURE S7 Genotyping profiles of four cooked meat samples amplified by this STR panel.
