# Supplementary material for: Development and Validation of a Novel Five-Dye Short Tandem Repeat Panel for Forensic Identification of 11 Species
Source: Front Genet. 2020 Sep 24;11:1005. doi: 10.3389/fgene.2020.01005 (PMC7541953; doi:10.3389/fgene.2020.01005)
Supplement: Supplementary file 1 [file Table_1.docx]

| Supplementary Table 1. Two kinds of mix DNA patterns with different mix ratios for mixture study. | | | | | | | | |  |  |  |
| --- | --- | --- | --- | --- | --- | --- | --- | --- | --- | --- | --- |
| Pattern I | 1 | Pig | Goat |  |  |  |  |  |  |  |  |
|  |  | 0.5000ng | 0.5000ng |  |  |  |  |  |  |  |  |
|  | 2 | Pig | Goat | Chicken |  |  |  |  |  |  |  |
|  |  | 0.3330ng | 0.3330ng | 0.3330ng |  |  |  |  |  |  |  |
|  | 3 | Pig | Goat | Chicken | Duck |  |  |  |  |  |  |
|  |  | 0.2500ng | 0.2500ng | 0.2500ng | 0.2500ng |  |  |  |  |  |  |
|  | 4 | Pig | Goat | Chicken | Duck | Cattle |  |  |  |  |  |
|  |  | 0.2000ng | 0.2000ng | 0.2000ng | 0.2000ng | 0.2000ng |  |  |  |  |  |
|  | 5 | Pig | Goat | Chicken | Duck | Cattle | Horse |  |  |  |  |
|  |  | 0.1667ng | 0.1667ng | 0.1667ng | 0.1667ng | 0.1667ng | 0.1667ng |  |  |  |  |
|  | 6 | Pig | Goat | Chicken | Duck | Cattle | Horse | Canine |  |  |  |
|  |  | 0.1429ng | 0.1429ng | 0.1429ng | 0.1429ng | 0.1429ng | 0.1429ng | 0.1429ng |  |  |  |
|  | 7 | Pig | Goat | Chicken | Duck | Cattle | Horse | Canine | Rat |  |  |
|  |  | 0.125ng | 0.125ng | 0.125ng | 0.125ng | 0.125ng | 0.125ng | 0.125ng | 0.125ng |  |  |
|  | 8 | Pig | Goat | Chicken | Duck | Cattle | Horse | Canine | Rat | Mouse |  |
|  |  | 0.1111ng | 0.1111ng | 0.1111ng | 0.1111ng | 0.1111ng | 0.1111ng | 0.1111ng | 0.1111ng | 0.1111ng |  |
|  | 9 | Pig | Goat | Chicken | Duck | Cattle | Horse | Canine | Rat | Mouse | Pigeon |
|  |  | 0.1000ng | 0.1000ng | 0.1000ng | 0.1000ng | 0.1000ng | 0.1000ng | 0.1000ng | 0.1000ng | 0.1000ng | 0.1000ng |
|  |  |  |  |  |  |  |  |  |  |  |  |
|  |  |  |  |  |  |  |  |  |  |  |  |
| Pattern II | 1 | Chicken | Pig |  |  |  |  |  |  |  |  |
|  |  | 3:1, 1:1, 1:3 | |  |  |  |  |  |  |  |  |
|  | 2 | Duck | Pig |  |  |  |  |  |  |  |  |
|  |  | 3:1, 1:1, 1:3 | |  |  |  |  |  |  |  |  |
|  | 3 | Cattle | Pig |  |  |  |  |  |  |  |  |
|  |  | 3:1, 1:1, 1:3 | |  |  |  |  |  |  |  |  |
|  | 4 | Goat | Pig |  |  |  |  |  |  |  |  |
|  |  | 3:1, 1:1, 1:3 | |  |  |  |  |  |  |  |  |
|  | 5 | Chicken | Cattle |  |  |  |  |  |  |  |  |
|  |  | 3:1, 1:1, 1:3 | |  |  |  |  |  |  |  |  |
|  | 6 | Duck | Cattle |  |  |  |  |  |  |  |  |
|  |  | 3:1, 1:1, 1:3 | |  |  |  |  |  |  |  |  |
|  | 7 | Chicken | Goat |  |  |  |  |  |  |  |  |
|  |  | 3:1, 1:1, 1:3 | |  |  |  |  |  |  |  |  |
|  | 8 | Duck | Goat |  |  |  |  |  |  |  |  |
|  |  | 3:1, 1:1, 1:3 | |  |  |  |  |  |  |  |  |
|  | 9 | Cattle | Goat |  |  |  |  |  |  |  |  |
|  |  | 3:1, 1:1, 1:3 | |  |  |  |  |  |  |  |  |
