# Supplementary figures and images for: Development and Validation of a Novel Five-Dye Short Tandem Repeat Panel for Forensic Identification of 11 Species
Source: Front Genet. 2020 Sep 24;11:1005. doi: 10.3389/fgene.2020.01005 (PMC7541953; doi:10.3389/fgene.2020.01005)

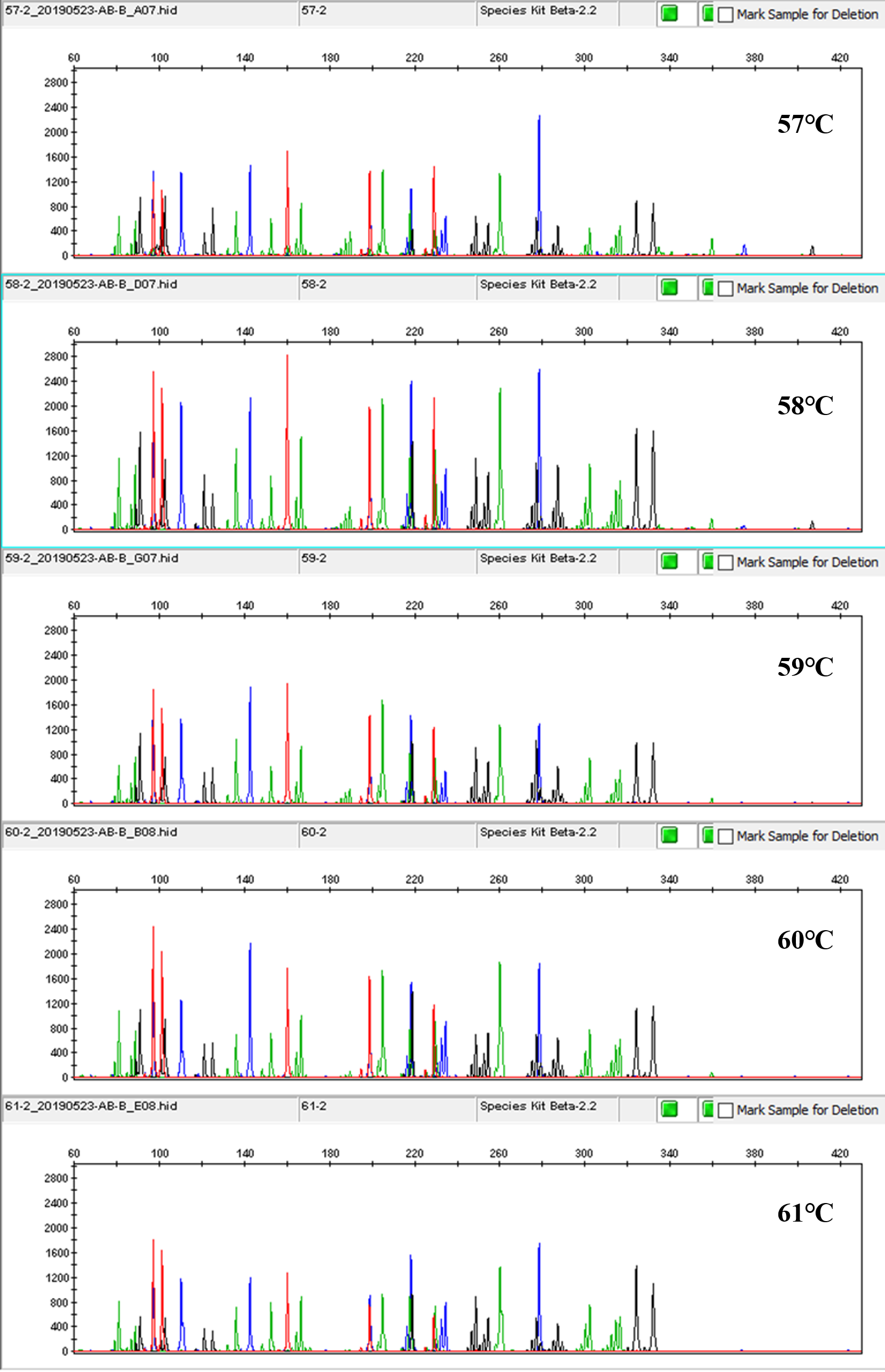

Supplement: FIGURE S1 — Genotyping profiles of annealing temperatures at 57, 58, 59, 60, and 61°C. [file Image_1.TIF]

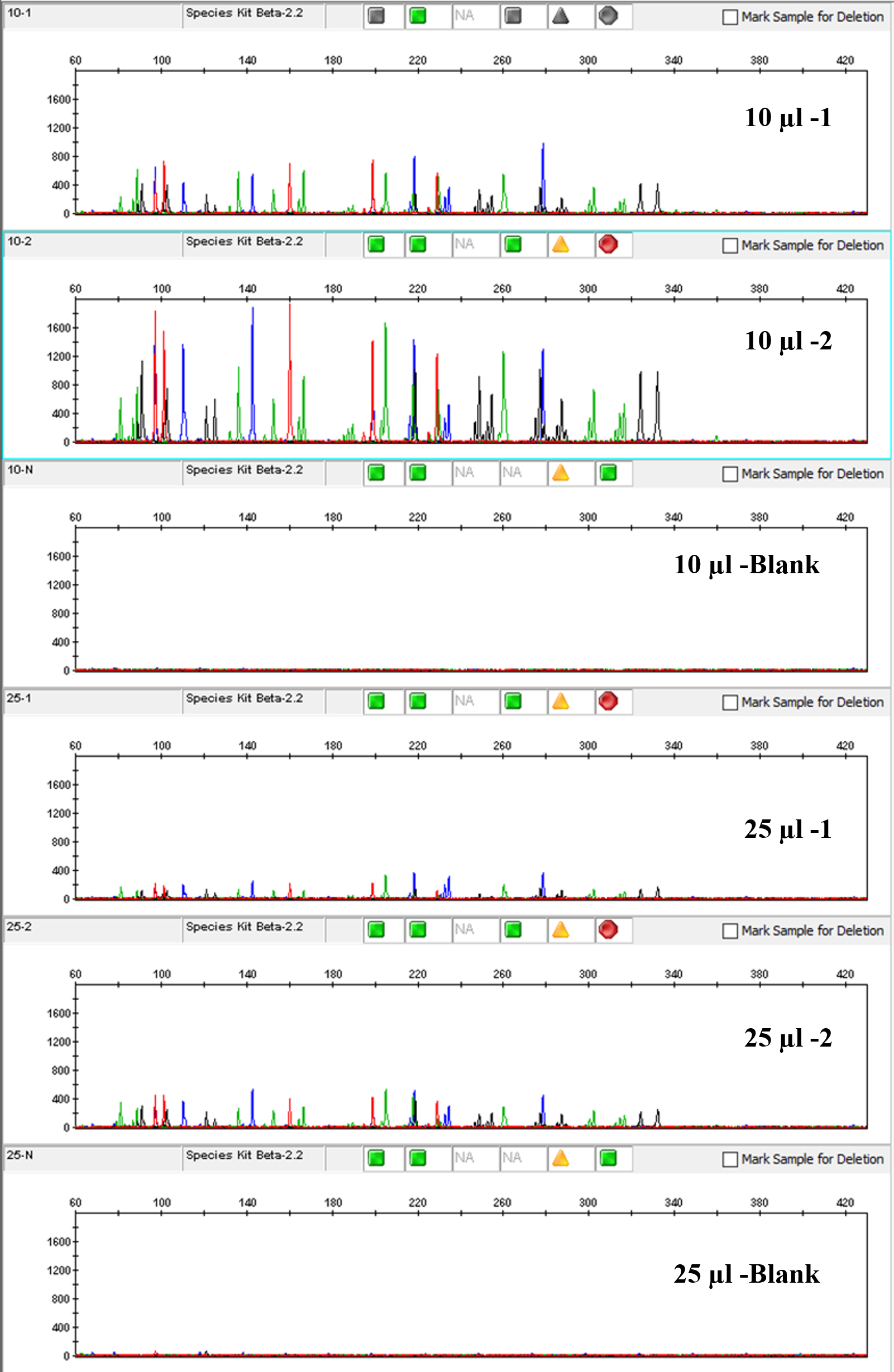

Supplement: FIGURE S2 — PCR efficiency studies of the two different reaction volume systems: 10 and 25 μl. [file Image_2.TIF]

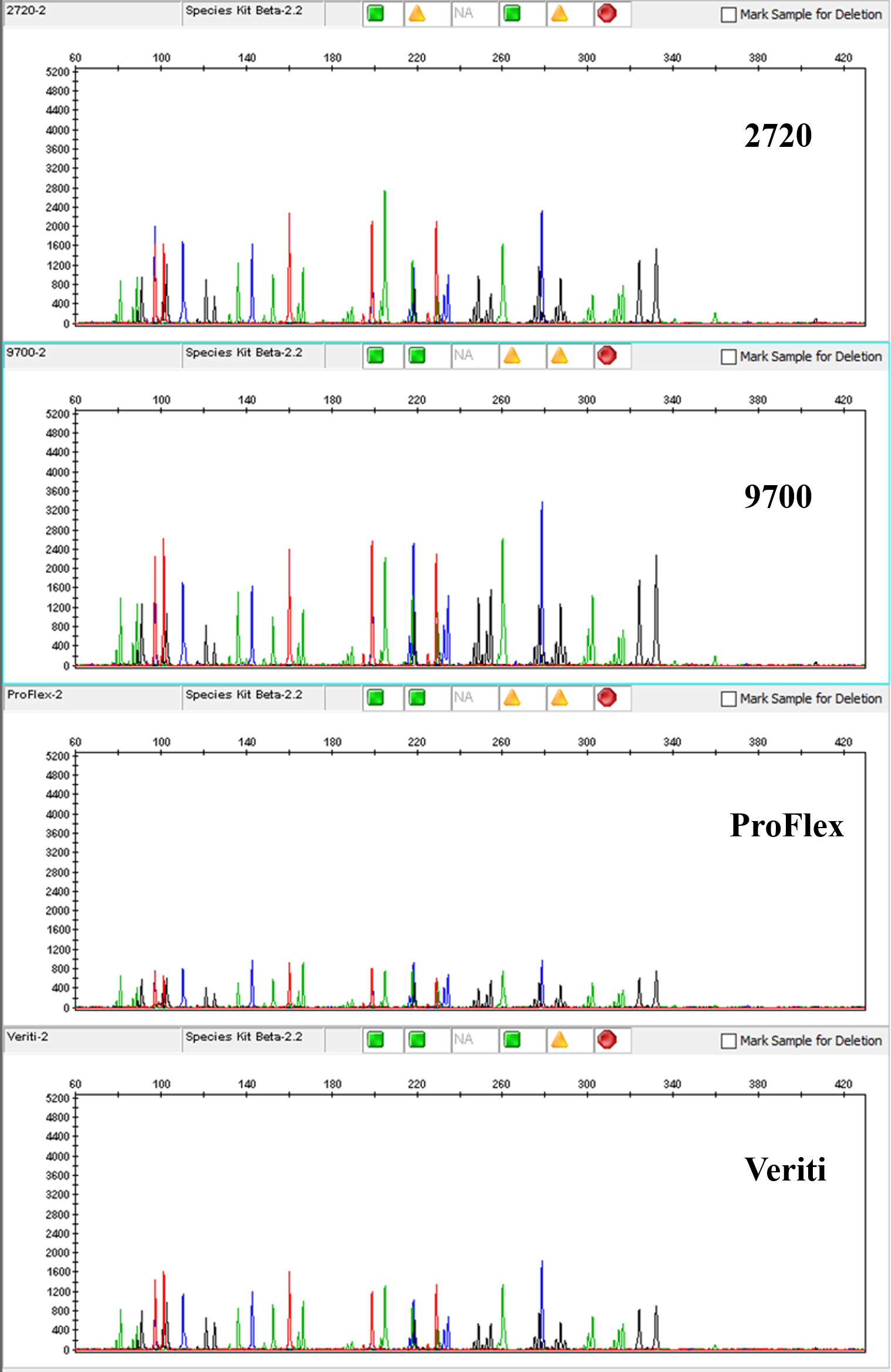

Supplement: FIGURE S3 — Genotyping profiles of PCR efficiency studies using four different types of PCR machines. [file Image_3.TIF]

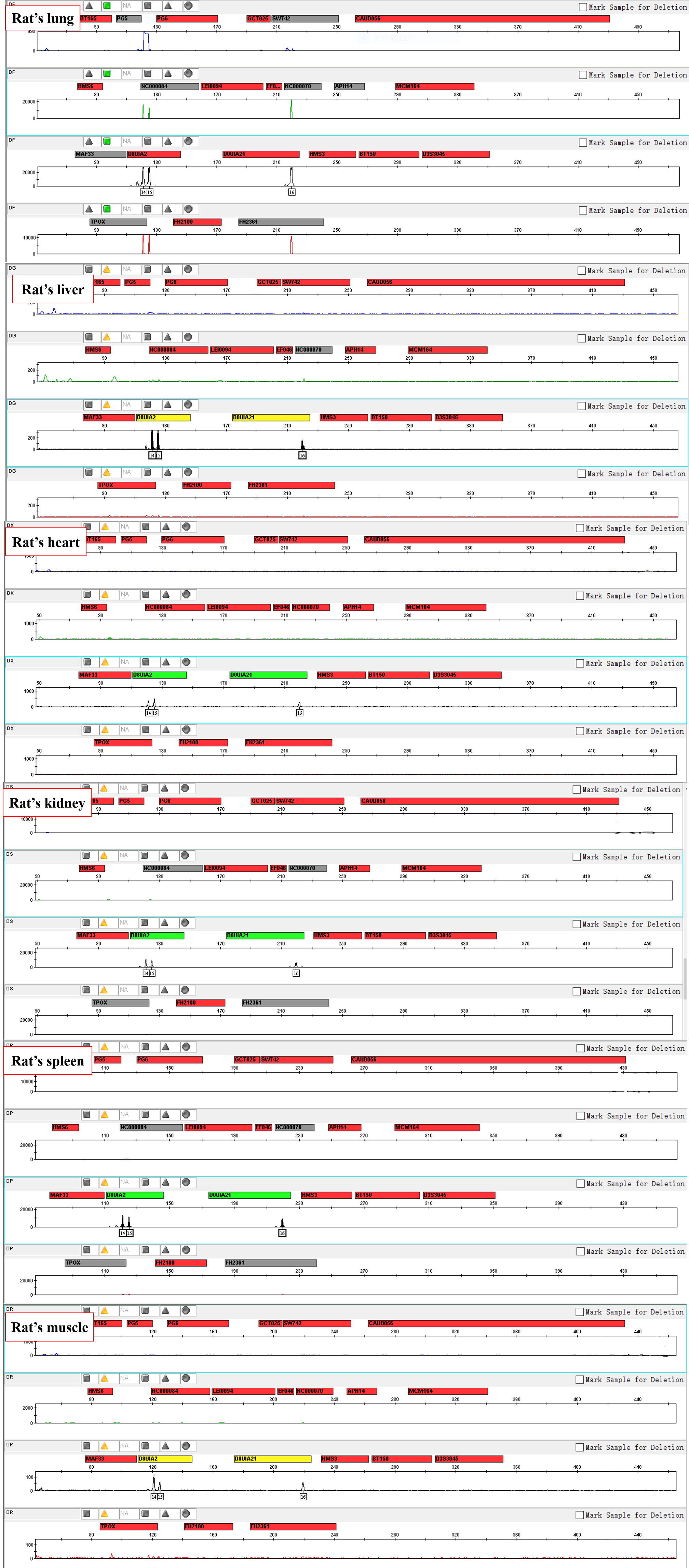

Supplement: FIGURE S4 — Genotyping profiles of DNA samples extracted from the liver, heart, spleen, lung, kidney, and muscle of the same SD rat. [file Image_4.TIF]

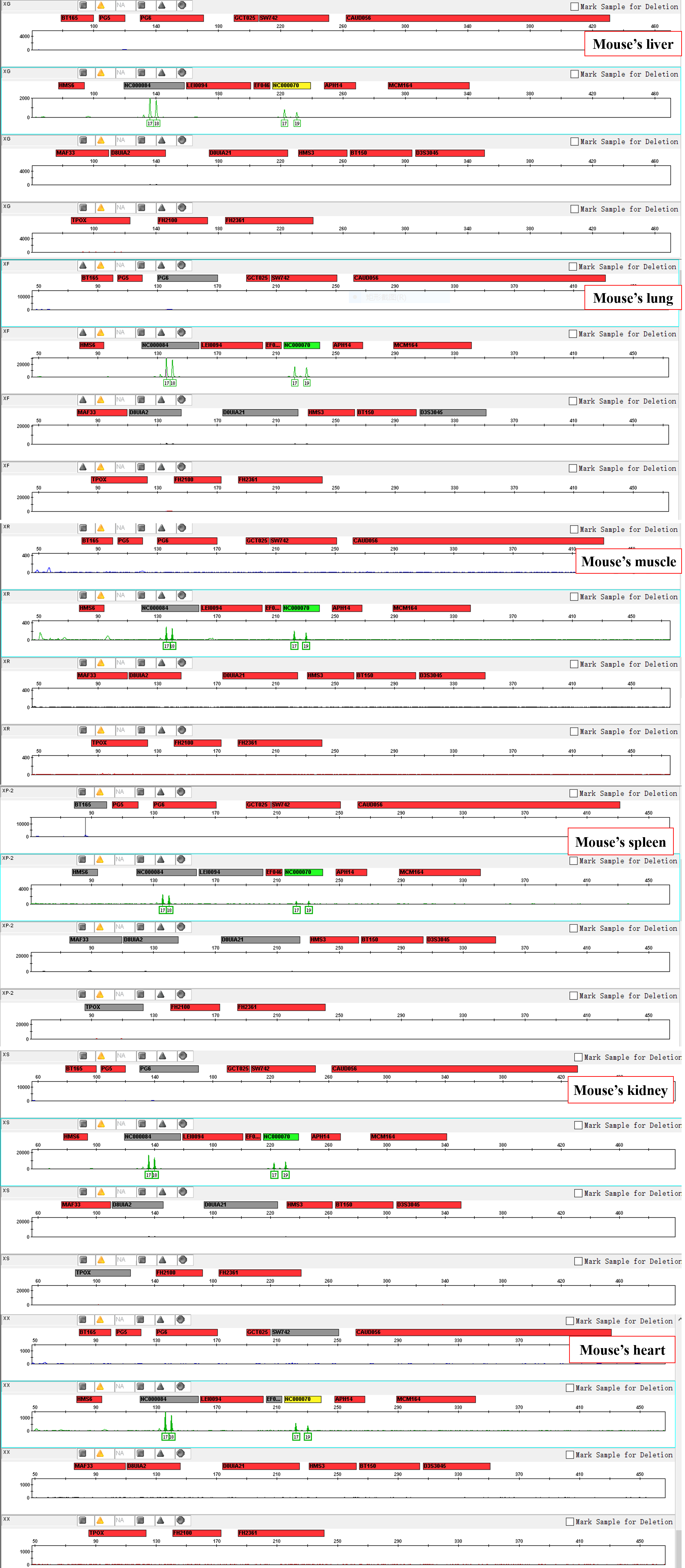

Supplement: FIGURE S5 — Genotyping profiles of DNA samples extracted from the liver, heart, spleen, lung, kidney, and muscle of the same Kunming mouse. [file Image_5.TIF]

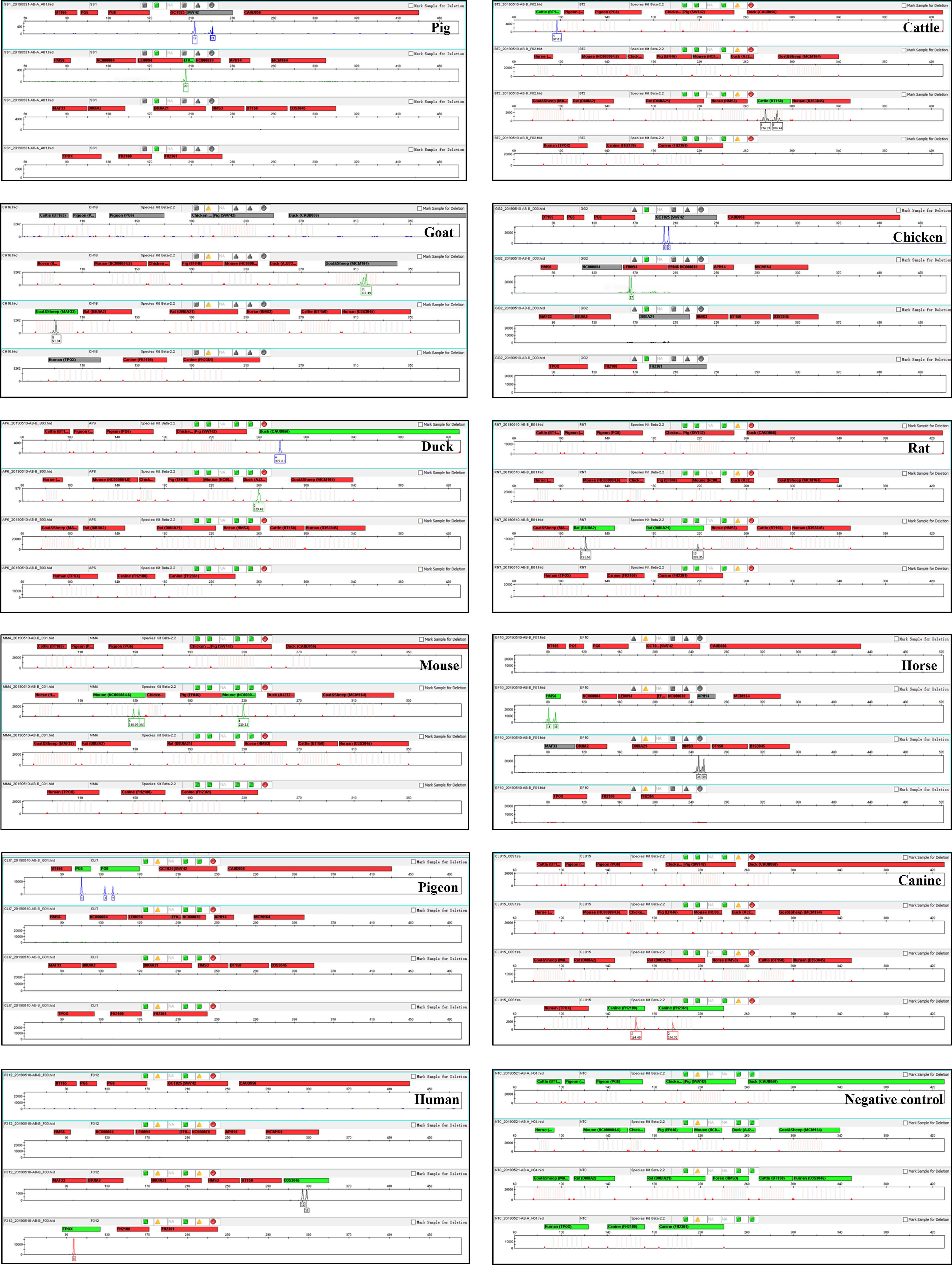

Supplement: FIGURE S6 — Genotyping profiles of species specificity studies on this 22-STR panel. [file Image_6.TIF]

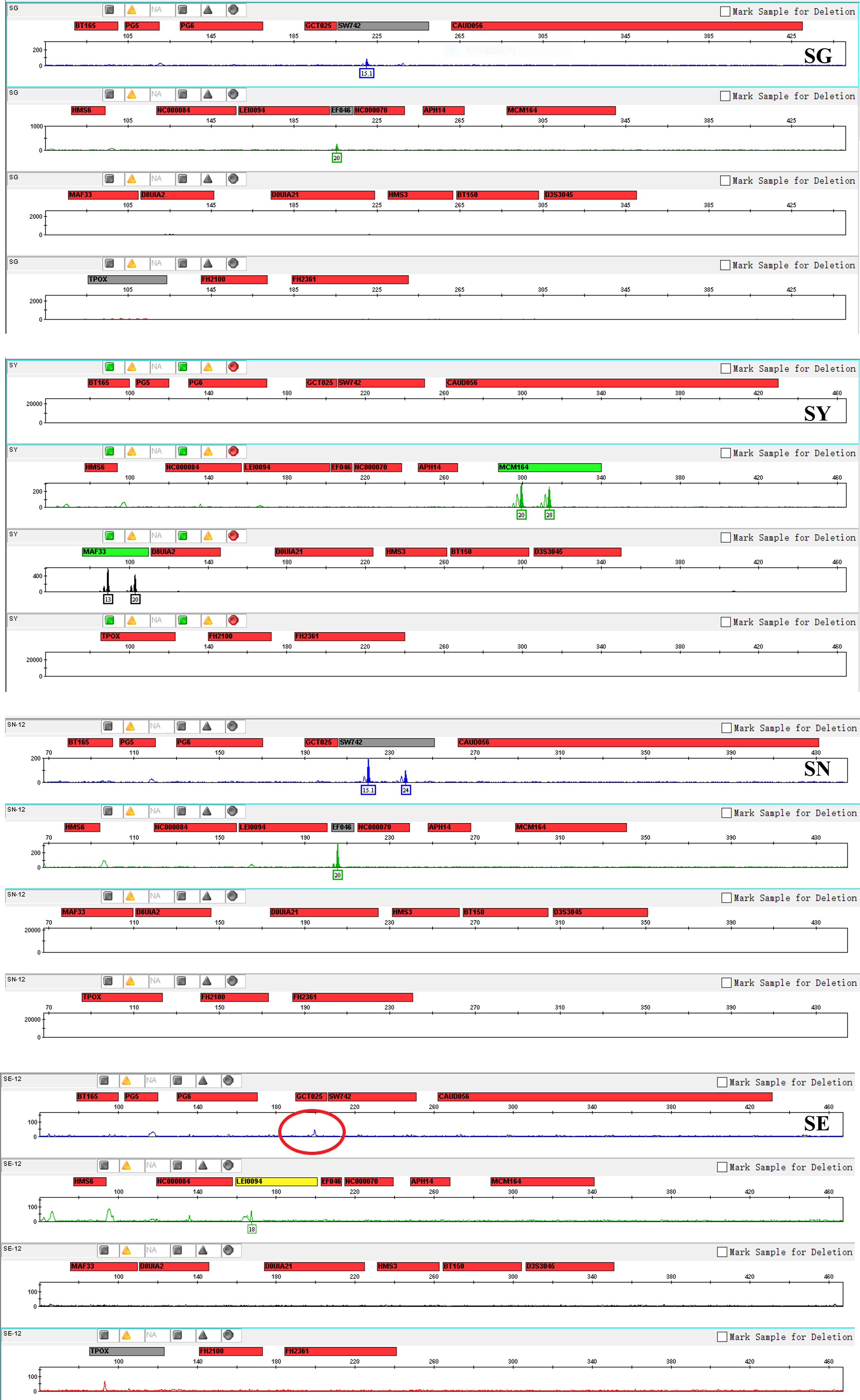

Supplement: FIGURE S7 — Genotyping profiles of four cooked meat samples amplified by this STR panel. [file Image_7.TIF]
